# Hooking the Self Onto the Past: How Positive Autobiographical Memory Retrieval Benefits People With Social Anxiety

**DOI:** 10.1177/21677026231195792

**Published:** 2023-11-15

**Authors:** David A. Moscovitch, Kendra White, Taylor Hudd

**Affiliations:** Department of Psychology and Centre for Mental Health Research and Treatment, University of Waterloo

**Keywords:** social anxiety, autobiographical memory, intervention, positivity, rewards

## Abstract

Do people with social anxiety (SA) benefit from positive memory retrieval that heightens self-relevant meaning? In this preregistered study, an analog sample of 255 participants with self-reported clinically significant symptoms of SA were randomly assigned to retrieve and process a positive social-autobiographical memory by focusing on either its self-relevant meaning (deep processing) or its perceptual features (superficial processing). Participants were then socially excluded and instructed to reimagine their positive memory. Analyses revealed that participants assigned to the deep processing condition experienced significantly greater improvements than participants in the superficial processing condition in positive affect, social safeness, and positive beliefs about others during initial memory retrieval and in negative and positive beliefs about the self following memory reactivation during recovery from exclusion. These novel findings highlight the potential utility of memory-based interventions for SA that work by “hooking” self-meaning onto recollections of positive interpersonal experiences that elicit feelings of social acceptance.

Human identity and self-knowledge depend on the ability to learn from the past. This ability, in turn, depends on autobiographical memory, a form of episodic memory for personal life events occurring at a specific place and time (Tulving, 1972). The autobiographical memory system is composed of a specialized neural network that revolves centrally around the hippocampus (Schacter et al., 2012). By directing the encoding, consolidation, and retrieval of specific past personal events, the autobiographical memory system derives critical information and meaning about the self from those events and guides goal-directed behavior based on self-knowledge (Conway, 2005; Conway & Pleydell-Pearce, 2000). This system evolved to support the adaptive use of mental simulation and, specifically, mental time travel to enable people to learn from past experiences as they navigate the social and emotional challenges of the present and future (Addis, 2020; M. Moscovitch et al., 2016; Schacter et al., 2007).

Given the established links between episodic memory and self-knowledge, it is no surprise that negative autobiographical memories play a prominent role in clinical disorders that are characterized by negative self-perception (R. T. Cohen & Kahana, 2022; D. A. Moscovitch et al., 2023). One such disorder is social anxiety disorder (SAD), a common and impairing problem in which people perceive themselves as being socially undesirable and fear that their perceived self-flaws will become exposed to critical others in social contexts (D. A. Moscovitch, 2009; D. A. Moscovitch et al., 2015).

These maladaptive views of self in SAD are strengthened and solidified by mental images and autobiographical memories of negatively interpreted personal experiences that are replayed repeatedly in the aftermath of anxiety-provoking social situations (Clark & Wells, 1995; Rapee & Heimberg, 1997). During mental replay, socially anxious people experience intrusive, vivid, recurrent, and distressing images and memories in which they envision themselves appearing or behaving in embarrassing or socially inappropriate ways (Chiupka et al., 2012; Hackmann et al., 1998, 2000; Hirsch et al., 2004; D. A. Moscovitch et al., 2011, 2018). Although these mental representations of the self are negatively biased and distorted, socially anxious people believe they are accurate reflections of how they appear to others (D. A. Moscovitch et al., 2013; Stopa, 2009). Experiences that are consistent with these negative self-views are encoded in memory as being personally meaningful and distressing (Morgan, 2010), and the meaning of these memories becomes encapsulated within the mental image as a reflection of socially anxious people’s core beliefs about self, others, and the world (Çili & Stopa, 2015; Holmes & Mathews, 2010; Reimer & Moscovitch, 2015). In this manner, for people with SAD, social memories consistent with negative views of self may come to be appraised as “self-defining”—affectively intense, repetitive, and vivid exemplars of self that inform their identity and frame their motivation for goal-directed behavior (Conway, 2005; Conway & Pleydell-Pearce, 2000; Krans et al., 2014; Sutherland & Bryant, 2005).

Considering the central role of autobiographical memory in sustaining negative self-beliefs that lie at the heart of SAD, intervention techniques have been developed to target negative self-defining memories. For example, imagery rescripting (IR) is a procedure in which patients are guided in three phases to adopt new perspectives on a past negative experience (see Arntz, 2012). In Phase 1 of IR, patients retrieve the original memory and relive it in detail from their younger selves’ first-person perspective. In Phase 2, they imagine their current selves intervening within the memory scene to fulfill the unmet needs of their younger selves, including providing compassion and support to their younger selves or assertively standing up for or protecting their younger selves (Romano et al., 2021). Finally, in Phase 3, they relive the memory again from the younger selves’ perspective but with the changes incorporated from Phase 2.

A single 90-min session of IR has been shown to improve social anxiety (SA) symptoms in patients with SAD and to achieve its effects, at least in part, through the process of “memory updating” (Wild & Clark, 2011). Indeed, recent treatment studies have shown that IR enables patients with SAD to incorporate more positive episodic details into memory recollection, reduce memory-related distress and vividness, and alter memory appraisals and meanings (Reimer & Moscovitch, 2015; Romano et al., 2021; Romano, Moscovitch, et al., 2020). Outside the clinic, similar memory updating effects have been documented when participants are instructed to relive past aversive experiences while purposely finding positive meaning in them (Speer et al., 2021). Thus, participants appear to benefit from memory-based techniques that guide them to “unhook” negative appraisals of themselves from past aversive experiences.

In contrast to the relatively large body of research on SA and negative autobiographical memory, there have been few studies on the relationship between SA and positive autobiographical memory. D. A. Moscovitch et al. (2018) found that participants with SAD recollected SA-provoking experiences in greater episodic detail than nonanxious control participants, but the two groups did not differ in the amount of episodic details recollected for non-anxiety-provoking social experiences; however, this study was designed to compare recall for aversive versus nonaversive experiences rather than memories of subjectively positive or pleasurable social experiences per se. Moreover, research by Romano, Tran, and Moscovitch (2020) found that high-trait SA was associated with inhibited recall of positive social information, but this study examined episodic memory for hypothetical scenarios rather than personal autobiographical memories. Stopa and Jenkins (2007) found that positive autobiographical memories were retrieved more slowly than negative autobiographical memories when participants were instructed to hold a negative image in mind during a speech task, but this study focused on the effects of self-imagery on the retrieval of memories, so the nature and impact of positive memory retrieval itself was not subjected to further analysis. Several other studies have shown that people with high levels of trait SA, especially people with SAD, struggle to recollect elements of positive self-relevant feedback and to update self-concepts in line with such feedback (Beltzer et al., 2019; Everaert et al., 2018; Glazier & Alden, 2019; Hopkins et al., 2021; Koban et al., 2017), but none of these studies focused specifically on the nature or impact of positive-autobiographical-memory retrieval.

Although there has been little to no research on the benefits of positive-autobiographical-memory retrieval in people with high trait SA, experimental studies on positive memory retrieval in healthy and depressed adults support its potential utility for people with high SA. First, research has shown that positive memory recall is more effective than neutral memory recall at engaging neural reward centers and dampening negative affective and physiological responsivity to acute stress (Speer & Delgado, 2017; Speer et al., 2014). Second, studies of positive memory retrieval in people with depression have demonstrated that recalling positive experiences in combination with amygdala neurofeedback boosted emotional processing of positive stimuli and enhanced feelings of happiness and pleasure (Young et al., 2016, 2017), although combining positive memory retrieval with neurofeedback prevented researchers from drawing clear conclusions about the effects of positive memory retrieval per se. Other studies have found that people with depression might benefit from intentional positive memory recall only under certain processing conditions—for example, if they adopt a self-reflective or concrete mode of processing rather than a self-evaluative or abstract mode of processing when retrieving the memory (Joormann et al., 2007; Watkins, 2008; Werner-Seidler & Moulds, 2012). Because SA and depression share overlapping features and are often comorbid with one another (see Rozen et al., 2022), it is possible that positive-memory-retrieval techniques with specific processing instructions may offer similar therapeutic benefits to people with SA, even in the absence of amygdala neurofeedback. Indeed, clinical interventions that guide high SA individuals to recognize and savor positive experiences have been shown to promote social connectedness, increase positive affect, and reduce negative affect (Taylor, Pearlstein, et al., 2020). However, current psychotherapeutic approaches to targeting positivity deficits in the treatment of emotional disorders have generally focused on comprehensive, multisession treatment protocols for mixed samples of anxious and depressed patients (Craske et al., 2016, 2019; Taylor et al., 2017). To the best of our knowledge, no prior studies have specifically isolated and tested the conditions under which positive autobiographical memory retrieval could benefit socially anxious individuals.

In designing the current study, we imagined that positive memory retrieval coupled with deep, self-relevant memory processing may be beneficial for socially anxious individuals in several ways. First, we reasoned that its benefits could resemble those of other brief positive psychology interventions that have become popular in recent years, which are conceptualized as purposeful strategies that can be self-administered in daily life for the purpose of temporarily boosting subjective psychological well-being (Bolier et al., 2013; Bryant et al., 2005; Meyers et al., 2013; Miguel-Alvaro et al., 2021; Sin & Lyubomirsky, 2009). Second, we theorized that positive memory retrieval could be beneficial if used as a type of stress inoculation strategy (Meichenbaum & Deffenbacher, 1988; Saunders et al., 1996) to enhance resistance to stress by preventing or mitigating the immediate impact of negative life experiences, such as rejection or exclusion (Hudd & Moscovitch, 2021; Taylor, Tsai, & Smith, 2020). Finally, we speculated that positive memory retrieval could function as a type of psychological repair strategy that could help facilitate resilient recovery from stressful experiences (Campbell-Sills et al., 2006; Fiksdal et al., 2019) that enables individuals to “bounce back” effectively from a painful or anxiety-provoking event (Hudd & Moscovitch, 2020).

To investigate these possibilities, we preselected an analog sample of participants with clinically significant self-reported levels of SA symptoms and instructed them to retrieve and write about a personal positive memory in which they felt socially connected, valued, or accepted. Participants were randomly assigned to process their positive memory either deeply by writing about its meaning in relation to the self or superficially by writing about its perceptual features. All participants were subsequently exposed to a distressing exclusion task that threatened their sense of belongingness. Following exclusion, they were instructed to reactivate their positive memory and focus on the details that had been retrieved during the writing task to which they had been assigned. Repeated self-report measures of state affect, social safeness, and beliefs about self and others were collected at baseline, initial retrieval, exclusion, and recovery.

We hypothesized that the benefits of positive memory retrieval would be especially apparent for socially anxious participants assigned to process their memory in a deep, self-relevant manner compared with participants assigned to process their memory in a shallow, superficial manner. Specifically, we predicted that compared with shallow processing, deep processing of the memory would more effectively (a) improve participants’ mood, felt social safeness, and beliefs about self and others at initial memory retrieval relative to baseline; (b) provide stronger protection against the negative effects of exclusion; and (c) facilitate more resilient recovery from threatened belongingness needs when reimagining their positive memory in the aftermath of exclusion.

## Transparency and Openness

### Preregistration

Hypotheses, methods, and data analytic plan were preregistered on the OSF website at https://osf.io/jve6h. Changes or deviations from the analytic plan are described below.

### Data, materials, code, and online resources

The final data set and syntax are publicly available on the OSF website at https://osf.io/zprd6/. Supplemental Material is available online.

### Reporting

We report how we determined our sample size, all data exclusions, all manipulations, and all measures in the study.

### Ethical approval

All study procedures were approved by the Human Research Ethics Board at the University of Waterloo and were conducted in accordance with the provisions of the World Medical Association Declaration of Helsinki.

## Method

### Participants

We administered the Social Phobia Inventory (SPIN; Connor et al., 2000), a standardized measure of SA symptoms, to all undergraduate students in the psychology research participation pool at our institution at the start of term. Following Connor et al.’s (2000) recommendation that scores of 19 or above on the SPIN are likely to reflect clinically significant levels of SA, only undergraduates with SPIN scores of 19 and above were invited to participate in the present study.

In the absence of prior work on the effects of positive memory retrieval in SA with established effect sizes to guide formal power analyses, a priori sample-size estimates were based on Brysbaert’s (2019) criterion that at least 100 participants per condition should be recruited to ensure sufficient power to detect a medium effect size with α of .05 and power of .8. For more information, see the Supplemental Material. After we excluded invalid data (described below), the final study sample consisted of 255 high SA participants. Table 1 summarizes participants’ demographic characteristics across conditions.

**Table 1.** Characteristics of the Study Sample Overall and in Each Condition

	Deep processing (*n* = 125)	Superficial processing (*n* = 130)	Overall (*N* = 255)
Age in years, *M* (*SD*)	20.12 (3.98)	19.93 (3.18)	20.02 (3.59)
Gender (%)			
Male	19.2	12.3	15.7
Female	77.6	85.4	81.6
Transgender: male to female	0.8	0.0	0.4
Transgender: female to male	0.0	0.0	0.0
Nonbinary	1.6	3.1	2.4
Other^a^	1.6	0.0	0.8
Prefer not to respond	0.0	0.0	0.0
Ethnic/cultural background (%)^b^			
First Nations/Métis/Inuit	0.0	0.8	0.4
Arab/West Asian/North African	4.8	6.2	5.5
Black/Afro-Caribbean/African	1.6	3.1	2.4
East Asian	21.6	16.9	19.2
Latin American	2.4	1.5	2.0
South Asian	20.0	23.8	22.0
Southeast Asian	8.8	6.9	7.8
White/European	48.0	41.5	44.7
None of the above^c^	0.8	2.3	1.6
Prefer not to respond	3.2	1.5	2.4

a Participants who selected “Other” were permitted to type in their own open-ended responses, which included “queergender” and “she/he/they anything.”

b Participants were instructed to “select all that apply.”

c Participants who selected “None of the Above” were permitted to type in their own open-ended responses, which included “Persian,” “Iraqi Arab,” and “Mixed Ethnicity.”

### Procedure

This online study was programmed in Qualtrics, data collection occurred from September 2021 to April 2022, and preregistration was completed in November 2021. Participants who provided consent and indicated they had access to a functioning audio system began the study by completing baseline state measures of the extended Positive and Negative Affect Schedule (PANAS), the Social Safeness and Pleasure Scale (SSPS), and the Brief Core Schema Scale (BCSS), as described in Measures, below. Then, all participants were instructed to recall a personal memory in which they felt accepted, connected, or valued by others. Participants who indicated they were unable to retrieve such a memory were provided with a series of three prompts designed to aid in bringing such a memory to mind, which are described in the Supplemental Material. Participants who were unable to retrieve a memory at the initial retrieval point and after all three prompts were excluded from the study. Participants who were able to retrieve a memory provided a one-sentence summary of the memory.

A block randomizer in Qualtrics was then used to assign participants randomly to one of two conditions: deep or superficial processing. Participants in each condition engaged in four consecutive 3-min writing-task segments, for a total of 12 min. To ensure strong engagement, task instructions were always presented both in writing and simultaneously via audio recording. The type of information that participants were instructed to focus on and write about in each segment differed between conditions; instructions in the deep processing condition guided participants to connect the meaning of the memory to their sense of self, and instructions in the superficial processing condition guided participants to recount perceptual and phonological details related to the memory scene. Illustrative examples of memory descriptions and narratives across conditions are provided in Table 2. At the conclusion of the 12-min writing task, participants within both conditions were readministered the same three questionnaires initially completed at baseline (extended PANAS, SSPS, BCSS).

**Table 2.** Illustrative Examples of Positive Memory Descriptions and Written Processing Narratives Across Conditions

Superficial processing condition		Deep processing condition	
Brief description	Written processing narrative	Brief description	Written processing narrative
Mall trip with my best friend	We are in the cafeteria, sitting in one of the booths, eating fries, talking, laughing enjoying each other’s company, taking videos/pictures together, creating memories, walking around, watching people walk by. It was in the afternoon, just me and my best friend. I’m wearing a green dress with black pants, she’s wearing a white shirt with black pants and gold jewelry. I can see the table, the fries (rectangular and yellow), the vegetables cut into cubes (green and red), and the rectangle headboard in the booth.	When I was little, my dad told me he’d always be proud of me after we finished watching a movie together	I was very young, so it felt like any other thing back then. I was happy. I was thinking that was good to know. I also thought that it was a bit weird to me that you’d have to tell someone that. I was happy. I didn’t behave in anyway differently than I normally would have at that age. I was young and did not know much about the world as I didn’t understand why that was something that needed to be said. It was my dad so I felt connected to him. It means that my dad is proud of me. It also means that I was a good person, probably still am. This means that I should trust myself more and trust who I am.
I came out to my brother as gay, then he accepted me with zero hesitance	I am outdoors with my brother, it’s at night. We were chatting. We were sitting on the sidewalk in between two buildings. The building next to us had a sort of shelf/hood we were under. I think my hair was short then. It is 9 pm. there are probably 5 people in total who are walking around the parking lot. They were all strangers. We didn’t know or pay attention to any of them. I was chatting with my brother so I never really looked at those strangers. I am holding my phone. I see the colors black and orange when I recall this event. It is very humid and the weather didn’t feel pleasant, but that’s okay because we are used to the weather.	I was crossed in love, and a good friend stayed with me until late	I was walking on the street at night and felt deeply lonely. I called my friend and she encouraged me and talked with me all night, and I then felt that there’s someone who cares about me, which made me feel a sense of belonging. My needs were satisfied through communication. Talking about my problem to someone close to me reduced my feeling of solitude and not being understood. It shows my friends care about me and will support me when needed. Empathy showed in conversations allowed me to feel connected. Maybe I’m a good person for others to love and care for, and other people may think that I am a good person who is worthy of help. It means my life is not lonely and failure, and I can count on close people when I need them.

Note: Phonological associations made by participants in the final phase of the writing task in the superficial processing condition (for task instructions, see the Supplemental Material available online) were omitted from the illustrative examples provided in this table.

All participants then played a game of Cyberball, a virtual game of catch with three other online players who were represented as avatars on the screen. Before participants made their first pass, they were instructed to share one piece of information about themselves with the “other players” in the game (who were actually just preprogrammed avatars) about their favorite book or movie. Likewise, the other avatars shared information with the participant at the start of the game to increase the salience of the cover story that they were playing catch with real people. Cyberball was preprogrammed by our research team to ensure that all participants received the ball on only one tenth of the total throws, which has been shown in multiple studies to activate the perception of being excluded and elicit moderate feelings of distress (see Hudd & Moscovitch, 2021). Following the exclusion task, participants completed the same measures for the third time.

**Fig. 1.**
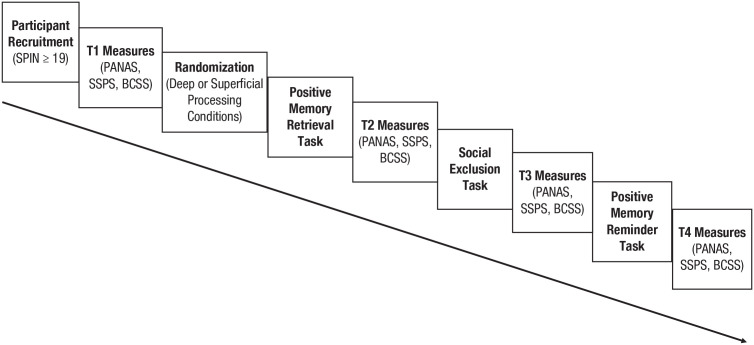
Outline of study procedures.

Immediately after completion of post-Cyberball measures, participants completed a 2-min memory-reminder task in which they were asked to recall their earlier memory, bring the image of it to mind, and summarize the main points of their earlier writings about the memory. Following the memory reminder task, participants completed the repeated measures for the fourth and final time. At this point, participants also completed a self-reported manipulation check assessing the extent to which they retrieved deeper or more superficial elements during the writing task, a Cyberball validity check assessing the extent to which they were able to participate fully in the task and were aware that they were thrown the ball more or less frequently than other players, a brief demographics questionnaire, and a short measure assessing their level of attention to and engagement with study procedures. Participants were then debriefed and remunerated with course credit. For a visual outline of study procedures, see Figure 1.

### Measures

#### Spin

SPIN (Connor et al., 2000) comprises 17 items measuring fear, avoidance, and psychological discomfort associated with social situations (e.g., “I avoid talking to people I don’t know”). Respondents rate the extent to which each of the 17 items troubled them over the past week using a scale ranging from 0 (*not at all*) to 4 (*extremely*). SPIN was administered before the study, during the prescreening, to preselect potentially eligible individuals for study participation who obtained a minimum score of 19 on SPIN, as described above. SPIN has shown strong psychometric properties, including good internal consistency, test-retest reliability, and convergent and divergent validity (Antony et al., 2006; Connor et al., 2000). This study provides additional support for this scale’s excellent internal consistency, as evidenced by a Cronbach’s α of .91.

#### The extended PANAS

The PANAS (Watson et al., 1988) is a 20-item self-report questionnaire that consists of 10 items assessing negative affect (e.g., distressed, nervous) and 10 items measuring positive affect (e.g., enthusiastic, excited). Critics have argued that the PANAS assesses only activated positive affect. To address this limitation and capture the wider scope of positive affect, Gilbert et al. (2008) developed an extended version of the PANAS that included an additional 15 emotion items encompassing relaxed (e.g., peaceful, calm) and safe/content (e.g., secure, warm) forms of positive affect. In the present study, we administered the extended state version of the PANAS, which instructs participants to rate the extent to which they feel each of the 35 items “right now” (i.e., at the moment of administration) using a 5-point scale ranging from 1 (*very slightly or not at all*) to 5 (*extremely*). The original PANAS has good psychometric properties, including test-retest reliability, internal reliability, and convergent and discriminant validity (Watson et al., 1988), but more research is needed to examine the psychometric properties of the extended PANAS. Internal consistency for the 10-item subscale assessing negative affect throughout the present study was strong, as indicated by Cronbach’s αs of .92 at baseline; .91 at Time 2 (T2), after the memory-retrieval writing task; .91 at Time 3 (T3), after exclusion; and .91 at Time 4 (T4), after memory recall. The extended 25-item positive affect scale also had strong internal consistency in the current study, as evidenced by Cronbach’s αs of .97, .97, .96, and .95 across Time 1 (T1) through T4, respectively.

#### SSPS

The SSPS (Gilbert et al., 2009) is an 11-item self-report measure that assesses the extent to which participants feel safe and secure within their interpersonal relationships. Social safeness was conceptualized by Gilbert et al. (2009) as an indicator of interpersonal warmth and connectedness related to the soothing-affiliation system. Participants are asked to indicate how much each item describes how they feel right now (e.g., “I feel a sense of belonging”) using a 5-point scale ranging from 0 (*almost never*) to 4 (*almost all the time*). Prior studies have shown that this scale demonstrates excellent reliability and construct and discriminant validity and that social safeness is distinct from positive or negative affect and can be conceptualized as a state-like construct that covaries over time with measures of received social support (Gilbert et al., 2009; Kelly et al., 2012). In the present study, Cronbach’s αs were .97, .98, .96, and .95 across T1 through T4, respectively.

#### BCSS

The BCSS (Fowler et al., 2006) is a 24-item self-report questionnaire that consists of negative and positive subscales that examines core beliefs about self and others. The scales evaluate four dimensions of self and other belief appraisals, including negative self (e.g., “I am unloved,” “I am a failure”), positive self (e.g., “I am valuable,” “I am good”), negative other (e.g., “Others are harsh,” “Others are unforgiving”), and positive other (e.g., “Others are trustworthy,” “Others are accepting”). Using a 4-point scale ranging from 0 = *slightly* to 4 = *totally*, participants are asked to rate their agreement with each of the 24 items based on how they feel right now. The BCSS has demonstrated good internal consistency, internal reliability, and concurrent and discriminant validity (Fowler et al., 2006). Although this scale has not been widely adopted for research outside the original validation study, its face validity appeared high for our desire to measure beliefs about self and others in the present study. Cronbach’s α values for negative beliefs about the self across the four time points were .87, .89, .85, and .86. Cronbach’s α values for positive beliefs about the self across the four time points were .93, .91, .89, and .89. Cronbach’s α values for negative beliefs about others across the four time points were .94, .93, .91, and .88. Cronbach’s α values for positive beliefs about others across the four time points were .93, .92, .91, and .87.

#### Manipulation check based on participant ratings

A 14-item self-report manipulation check was administered to measure the extent to which participants followed manipulation instructions during the memory-retrieval task using a 5-point rating scale ranging from 1 (*not at all*) to 5 (*extremely*). A single item assessed the extent to which participants were able to retrieve a relevant positive memory. Eight items measured the extent to which participants were able to deeply process their memory (e.g., “While reliving your memory, to what extent did you focus on your deepest thoughts and feelings?” “While reliving your memory, to what extent did you focus on what this experience says or means about you as a person?”), and these demonstrated strong internal consistency in the current study (α = .84). Five items assessed the extent to which participants reported engaging in superficial processing (e.g., “While reliving your memory, to what extent did you focus on where you were located in space and time within the memory scene?” “While reliving your memory, to what extent did you focus on the shapes, sizes, and/or colors of objects that may have been present in the environment within your memory?”), and these had satisfactory internal consistency (α = .80).

#### Manipulation check based on objective raters’ ratings of participants’ written narratives

Following the study, participants’ written narratives were collated into a randomized order on Qualtrics and rated one at a time by two research assistants (RAs) who were blind to the study purpose and manipulation condition from which each narrative was derived. RAs rated two items that we designed to correspond with deep and superficial processing outcomes, respectively: (a) “How much does the participant focus on describing the meaning of the recollected experience in relation to their sense of self (e.g., what the experience says about their life, their future, their relationships with others, and/or who they are as a person?)” and (b) “How much does the participant focus on describing the perceptual elements of the memory scene (e.g., who was there, what the people in the scene were doing/wearing/etc., what things looked like in the environment where the experience took place, etc.)?” Both items were rated on a 5-point scale ranging from 0 (*not at all*) to 4 (*extremely*). For information about RA training and additional ratings, see the Supplemental Material.

#### Cyberball validity check

Participants completed two items in which they rated the extent to which they were aware that they were thrown the ball more or less frequently than other players on a 5-point Likert scale ranging from 1 (*no, not at all*) to 5 (*yes, definitely*).

#### Overall attention and engagement check

A three-item measure was administered to assess how attentive and engaged participants were throughout the study on a 5-point scale ranging from 0 (*not at all*) to 4 (*a great deal*; e.g., “I was distracted while reading or answering questions”).

## Data Cleaning and Data Analytic Strategy

### Excluded data

A total of 284 participants were recruited for this study; 29 participants were excluded from analyses for either failing to provide consent for use of their data (*n* = 19) or failing to describe a memory that conformed with instructions to recall a positive social-autobiographical memory that occurred at a specific time and place (*n* = 10). Participants were also flagged for further inspection if they failed any self-reported and embedded attention checks, but no participants were ultimately excluded on this basis because all responses were deemed acceptable.

### Primary analyses

A series of 2 × 4 mixed-model omnibus analyses of variance (ANOVAs) were conducted with the between-subjects factor of condition (deep vs. superficial processing) and the within-subjects factor of time (T1, baseline; T2, after memory retrieval; T3, after exclusion; T4, after memory reminder) on measures of positive and negative affect, social safeness, and positive and negative beliefs about self and others.^1^ Main and interaction effects based on the Greenhouse-Geisser correction were reported whenever Mauchly’s test of sphericity was significant. Significant omnibus interaction effects for specific measures were followed up with three sets of 2 × 2 repeated measures ANOVAs to investigate the effects of condition on those measures at each phase of the study separately: at initial retrieval, in response to exclusion, and during recovery from social threat. Significant simple effects were probed with Bonferroni-corrected pairwise comparisons with control for Type 1 error, although our preregistered plan did not specify that such corrections would be performed. Independent-samples *t* tests were performed with bootstrapping (1,000 samples) to examine relative magnitudes of change scores across conditions in the presence of significant Time × Condition interaction effects, although bootstrapping was not specified in our preregistered plan. Interpretation of effect sizes followed J. Cohen’s (1988) recommendations, with partial η^2^ values of .01, .06, and .14 and *d* values of 0.20, 0.50, and 0.80 representing small, medium, and large effect sizes, respectively. Such benchmarks are commonly used to interpret effect sizes in psychological research but were not included specifically in our preregistered plan.

## Results

### Descriptive statistics and equivalence of SA symptoms across conditions

Means and standard deviations for outcome measures at each time point are presented in Table 3. There were no significant differences in SPIN scores between participants assigned to the superficial processing condition (*M* = 32.92, *SD* = 14.09) versus the deep processing condition (*M* = 35.06, *SD* = 12.42), *t*(252) = 1.283, *p* = .201, 95% confidence interval [CI] = [−1.15, 5.43].

**Table 3.** Descriptive Statistics for All Outcome Variables Across Time and Conditions

	Condition					
	Deep processing			Superficial processing		
	*N*	*M*	*SD*	*N*	*M*	*SD*
Negative affect						
T1	125	19.94	7.31	129	20.97	9.26
T2	124	17.85	6.72	129	19.17	8.38
T3	123	19.91	7.71	130	20.67	8.54
T4	124	17.80	6.82	128	19.32	8.29
Positive affect						
T1	124	58.46	15.54	127	61.52	20.05
T2	124	64.84	17.25	129	62.98	20.45
T3	124	53.15	17.22	129	55.90	22.07
T4	123	55.68	17.81	129	57.63	23.09
Social safeness						
T1	125	35.15	9.49	130	36.39	10.09
T2	124	39.19	9.70	129	38.16	10.59
T3	124	34.15	11.04	130	34.96	11.87
T4	122	36.97	10.15	130	36.80	11.54
Negative self-beliefs						
T1	125	12.47	4.65	129	12.71	5.14
T2	124	10.64	4.10	130	11.57	4.47
T3	123	11.78	4.82	130	12.26	5.15
T4	123	10.88	4.34	130	12.19	5.11
Positive self-beliefs						
T1	125	18.13	4.51	129	18.58	5.21
T2	124	19.67	4.70	130	19.42	5.21
T3	124	17.35	5.13	129	17.87	5.74
T4	123	18.75	5.25	130	18.38	5.52
Negative other beliefs						
T1	124	13.99	4.20	130	14.25	4.59
T2	124	12.66	3.92	130	13.73	5.08
T3	124	14.10	4.31	129	14.33	5.20
T4	123	12.85	4.09	130	13.72	5.43
Positive other beliefs						
T1	124	18.12	3.73	130	18.12	4.04
T2	124	19.70	4.20	130	18.62	4.26
T3	124	16.78	4.31	130	16.66	4.82
T4	123	18.07	4.45	130	17.87	4.55

Note: T1 = baseline; T2 = following manipulation and memory retrieval task; T3 = following exclusion task; T4 = following memory reminder task.

### Nature and frequency of memory recall

Of the final sample of 255 participants, 231 successfully retrieved a positive social autobiographical memory in which they felt accepted, connected, or valued in response to initial instructions, without any additional prompting. Of the 24 participants who initially reported being unable to retrieve an appropriate memory, 19 did so after one additional prompt, two did so after two additional prompts, and three did so after three additional prompts. We reviewed participants’ written descriptions to ensure that the memories that were retrieved conformed to required instructions—in other words, that they were true autobiographical memories of social experiences that occurred at a specific time and place and that were associated with feeling connected, valued, or accepted by others. As noted above, 10 participants were originally excluded from analyses on this basis. Although many participants whose data were included in the final analyses reported positive memories of engaging in pleasurable social activities, some participants reported positive memories of feeling supported and accepted by significant others during a difficult life event (see Table 2).

### Coder reliability

Two-way random-effects intraclass correlation coefficients (ICCs) were computed for absolute agreement of the average rating (because the average rating between the two raters was our intended unit of analysis). Results revealed ICC values of .883 and .885 for deep processing and superficial processing, respectively, which reflect “good” agreement (Koo & Li, 2016).

### Deep- and superficial-processing manipulation checks

#### Participant self-report measure

Consistent with expectations, the superficial processing manipulation check revealed significant differences between conditions, *t*(249) = −9.354, *p* ≤ .001, *d* = 1.18, such that participants assigned to the superficial processing condition (*M* = 13.01, *SD* = 3.55) reported a greater degree of superficial processing than participants assigned to the deepprocessing condition (*M* = 8.84, *SD* = 3.51). However, contrary to expectations, the extent to which participants reported engaging in deep processing did not differ significantly between conditions, *t*(242) = 1.669, *p* = .096, *d* = 0.21; means trended in the expected direction across the deep processing (*M* = 32.03, *SD* = 6.15) and superficial processing (*M* = 30.73, *SD* = 6.02) conditions.

#### Objective raters

Ratings of the extent to which narratives reflected superficial processing of perceptual features in the memory scene were significantly higher in the superficial processing relative to the deep processing condition, *t*(253) = −21.86, *M* difference = −1.71, *p* < .001, 95% CI = [−1.87, −1.56], *d* = 2.74; means were in the expected direction across the superficial (*M* = 2.54, *SD* = 0.64) and deep (*M* = 0.93, *SD* = 0.61) processing conditions. Conversely, the extent to which narratives reflected deep processing in relation to sense of self was rated as being significantly higher in the deep processing relative to the superficial processing condition, *t*(253) = 23.967, *M* difference = 2.14, *p* < .001, 95% CI = [1.97, 2.32], *d* = 2.74; means were in the expected direction across the deep (*M* = 2.87, *SD* = 0.64) and superficial (*M* = 0.72, *SD* = 0.78) processing conditions. Thus, objective ratings indicated that writing instructions across conditions successfully guided participants to process their memory narratives in a manner that was consistent with the experimental design, with very large but selective enhancements in either deep or superficial processing depending on the condition to which participants were assigned.

### Cyberball validity check

On average, participant ratings indicated that they perceived themselves as having received the ball less frequently than other players (*M* = 4.59, *SD* = 0.86) and not more frequently than other players (*M* = 1.37, *SD* = 0.84), suggesting the exclusion manipulation was successful.

### Primary analyses

#### Effects of condition and time on negative affect

Omnibus analyses demonstrated a significant change in negative affect over time, *F*(2.52, 624.34) = 15.625, *p* < .001, η_*p*_^2^ = .06. The main effect of condition was not significant, *F*(1, 248) = 1.617, *p* = .205, η_*p*_^2^ = .01, nor was the interaction between condition and time, *F*(2.52, 624.34) = 0.300, *p* = .790, η_*p*_^2^ = .001. Pairwise comparisons probing the significant main effect of time revealed that negative affect decreased significantly after initial positive memory retrieval compared with baseline, *M* difference = 1.91, *p* < .001, 95% CI = [1.02, 2.80]; increased significantly after the social exclusion task, *M* difference = −1.73, *p* < .001, 95% CI = [−2.81, −0.64]; and decreased significantly once again after the positive memory-reminder task, *M* difference = 1.75, *p* < .001, 95% CI = [0.79, 2.71].

#### Effects of condition and time on positive affect

Omnibus analyses revealed a significant change in positive affect over time, *F*(2.80, 688.79) = 43.197, *p* < .001, η_*p*_^2^ = .15. The main effect of condition was not significant, *F*(1, 246) = .548, *p* = .460, η_*p*_^2^ = .002. However, there was a significant interaction between condition and time, *F*(2.80, 688.79) = 3.260, *p* = .024, η_*p*_^2^ = .01. Follow-up 2 × 2 tests were conducted to probe the significant interaction effect at each phase of the study separately.

At initial memory retrieval, there was a significant Time × Condition interaction, *F*(1, 248) = 7.824, *p* = .006, η_*p*_^2^ = .03. Pairwise comparisons demonstrated that positive affect increased significantly after initial memory retrieval compared with baseline in the deep processing condition, *M* difference = −6.11, *p* < .001, 95% CI = [−8.44, −3.79], whereas for participants in the superficial processing condition, positive affect did not change significantly after initial memory retrieval compared with baseline, *M* difference = −1.48, *p* = .204, 95% CI = [−3.77, 0.81]. The magnitude of these differences in change scores from T1 to T2 varied significantly across conditions; there was a more robust increase at initial retrieval relative to baseline in the deep processing condition relative to the superficial processing condition, *t*(248) = −2.80, *M* difference = −4.63, *p* = .004, 95% CI = [−7.88, −1.24], *d* = 0.35. At exclusion, there was also a significant Time × Condition interaction, *F*(1, 250) = 5.660, *p* = .018, η_*p*_^2^ = .02. Pairwise comparisons demonstrated that positive affect decreased significantly after exclusion compared with initial retrieval in both the deep processing condition, *M* difference = 11.69, *p* < .001, 95% CI = [8.90, 14.47], and the superficial processing condition, *M* difference = 6.96, *p* < .001, 95% CI = [4.22, 9.71]. The magnitude of these differences in change scores from T2 to T3 varied significantly across conditions; there was a more robust decrease in positive affect following exclusion relative to initial retrieval in the deep processing condition than in the superficial processing condition, *t*(250) = 2.38, *M* difference = 4.72, *p* = .015, 95% CI = [1.11, 8.53], *d* = 0.30. At recovery, there was no Time × Condition interaction, *F*(1, 250) = .215, *p* = .643, η_*p*_^2^ = .000. Participants in the deep processing condition reported significant elevations in positive affect from exclusion to recovery, *M* difference = −2.55, *p* = .044, 95% CI = [−5.02, −0.07]. Participants in the superficial processing condition also reported an increase in positive affect from exclusion to recovery, but these changes were not statistically significant, *M* difference = −1.73, *p* = .161, 95% CI = [−0.66, 4.62].

#### Effects of condition and time on social safeness

Omnibus analyses revealed a significant change in social safeness over time, *F*(2.69, 669.34) = 37.375, *p* < .001, η_*p*_^2^ = .13. The main effect of condition was not significant, *F*(1, 249) = 0.009, *p* = .925, η_*p*_^2^ = .000, but there was a significant Condition × Time interaction effect, *F*(2.69, 669.34) = 3.142, *p* = .030 η_*p*_^2^ = .01. Follow-up 2 × 2 tests were conducted to probe the significant interaction effect at each phase of the study separately.

At initial retrieval, there was a significant Time × Condition interaction, *F*(1, 251) = 9.282, *p* = .003, η_*p*_^2^ = .04. Pairwise comparisons demonstrated that social safeness increased significantly after initial memory retrieval compared with baseline in both the deep processing condition, *M* difference = −4.02, *p* < .001, 95% CI = [−5.04, −2.99], and the superficial processing condition, *M* difference = −1.79, *p* < .001, 95% CI = [−2.80, −0.78]. The magnitude of these differences in change scores from T1 to T2 varied significantly across conditions. The deep processing condition was associated with a more robust increase in social safeness at initial retrieval relative to baseline than the superficial processing condition, *t*(251) = −3.05, *M* difference = −2.23, *p* = .003, 95% CI = [−3.66, −0.79], *d* = 0.38.

At exclusion, there was also a significant Time × Condition interaction, *F*(1, 251) = 4.344, *p* = .038, η_*p*_^2^ = .02. Pairwise comparisons demonstrated that social safeness decreased significantly after exclusion compared with initial retrieval in both the deep processing condition, *M* difference = 5.04, *p* < .001, 95% CI = [3.76, 6.32], and the superficial processing condition, *M* difference = 3.15, *p* < .001, 95% CI = [1.90, 4.40]. The magnitude of these differences in change scores from T2 to T3 varied significantly across conditions. There was a larger decrease in social safeness following exclusion relative to initial retrieval in the deep processing condition than in the superficial processing condition, *t*(250) = 2.08, *M* difference = 1.89, *p* = .038, 95% CI = [0.10, 3.68], *d* = .26.

At recovery, there was no Time × Condition interaction, *F*(1, 250) = 1.241, *p* = .266, η_*p*_^2^ = .01. Participants reported significant elevations in social safeness compared with exclusion in both the deep processing condition, *M* difference = −2.66, *p* < .001, 95% CI = [−3.71, −1.62], and the superficial processing condition, *M* difference = −1.84, *p* < .001, 95% CI = [−2.85, −0.82].

#### Effects of condition and time on negative self-beliefs

Omnibus analyses revealed a significant change in negative self-beliefs over time, *F*(2.58, 641.90) = 21.924, *p* < .001, η_*p*_^2^ = .08. The main effect of condition was not significant, *F*(1, 249) = 1.629, *p* = .203, η_*p*_^2^ = .01, but again, there was a significant interaction between condition and time, *F*(2.58, 641.90) = 3.073, *p* = .03, η_*p*_^2^ = .01. Follow-up 2 × 2 tests were conducted to probe the significant interaction effect at each phase of the study separately.

At initial retrieval, the Time × Condition interaction was not significant, *F*(1, 251) = 2.683, *p* = .103, η_*p*_^2^ = .01. Pairwise comparisons demonstrated that negative beliefs about the self decreased significantly and comparably after initial memory retrieval compared with baseline in both the deep processing condition *M* difference = 1.83, *p* < .001, 95% CI = [1.26, 2.40], and the superficial processing condition, *M* difference = 1.17, *p* < .001, 95% CI = [0.62, 1.73].

At exclusion, the Time × Condition interaction was not significant, *F*(1, 251) = 1.189, *p* = .277, η_*p*_^2^ = .01. Pairwise comparisons demonstrated that negative self-beliefs increased significantly and comparably after exclusion compared with initial retrieval in both conditions, including the deep processing condition, *M* difference = −1.11, *p* < .001, 95% CI = [−1.66, −0.57], and the superficial processing condition, *M* difference = −0.69, *p* = .011, 95% CI = [−1.22, −0.16].

**Fig. 2.**
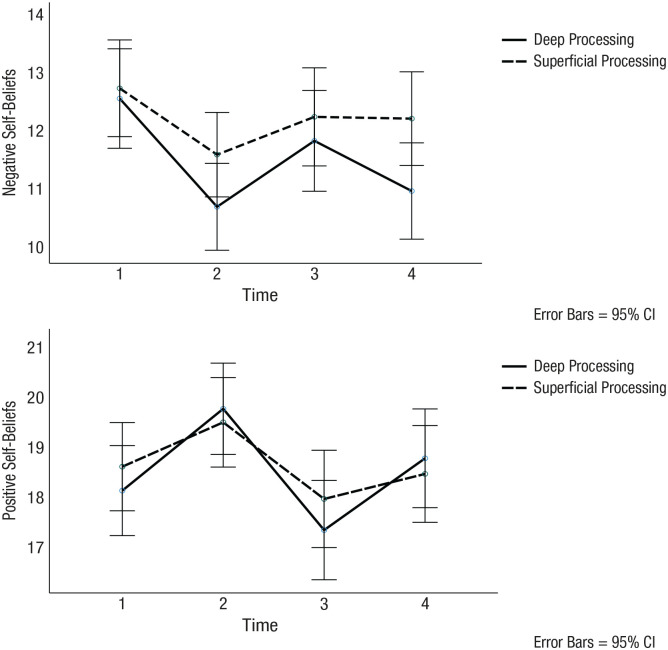
Changes over time in (top) negative and (bottom) positive self-beliefs. T1 = baseline; T2 = following manipulation and memory-retrieval task; T3 = following exclusion task; T4 = following memory-reminder task; solid line = deep-processing condition; dotted line = superficial-processing condition; error bars = 95% confidence intervals.

At recovery, the Time × Condition interaction was significant, *F*(1, 251) = 4.926, *p* = .027, η_*p*_^2^ = .02. Participants in the deep processing condition experienced significant reductions in negative self-beliefs compared with exclusion, *M* difference = 0.89, *p* < .001, 95% CI = [0.37, 1.41]. In contrast, participants in the superficial processing condition experienced no changes in negative self-beliefs at recovery compared with exclusion, *M* difference = 0.07, *p* = .787, 95% CI = [−0.44, 0.57]. The magnitude of these differences in change scores from T3 to T4 varied significantly across conditions. There was a larger decrease in negative self-beliefs at recovery relative to exclusion in the deep processing versus the superficial processing condition, *t*(250) = 2.22, *M difference* = 0.82, *p* = .03, 95% CI = [0.14, 1.58], *d* = 0.28. At the final recovery time point, participants in the deep processing condition rated the strength of their negative self-beliefs significantly lower than participants in the superficial processing condition, *M* difference = 1.29, *p* = .032, 95% CI = [0.11, 2.47]. These effects are shown in Figure 2.

#### Effects of condition and time on positive self-beliefs

Omnibus analyses revealed a significant change in positive self-beliefs over time, *F*(2.85, 709.73) = 33.934, *p* < .001, η_*p*_^2^ = .12. The main effect of condition was not significant, *F*(1, 249) = .042, *p* = .837, η_*p*_^2^ = .00, but there was a significant Condition × Time interaction, *F*(2.85, 709.73) = 3.040, *p* = .031, η_*p*_^2^ = .01. Follow-up 2 × 2 tests were conducted to probe the significant interaction effect at each phase of the study separately.

At initial retrieval, the Time × Condition interaction was not significant, *F*(1, 251) = 3.163, *p* = .077, η_*p*_^2^ = .01. Pairwise comparisons demonstrated that positive beliefs about the self increased significantly after initial memory retrieval compared with baseline in both the deep processing condition, *M* difference = −1.55, *p* < .001, 95% CI = [−2.08, −1.02], and the superficial processing condition, *M* difference = −0.88, *p* = .001, 95% CI = [−1.40, −0.36].

At exclusion, the Time × Condition interaction was not significant, *F*(1, 251) = 3.341, *p* = .069, η_*p*_^2^ = .01. Pairwise comparisons demonstrated that positive self-beliefs decreased significantly and comparably after exclusion compared with initial retrieval in both conditions, including the deep processing condition, *M* difference = 2.32, *p* < .001, 95% CI = [1.72, 2.92], and the superficial processing condition, *M* difference = 1.54, *p* < .001, 95% CI = [0.95, 2.13].

At recovery, the Time × Condition interaction was significant, *F*(1, 250) = 6.723, *p* = .010, η_*p*_^2^ = .03. Participants experienced significant increases in positive self-beliefs at recovery compared with exclusion in both the deep processing condition, *M* difference = −1.39, *p* < .001, 95% CI = [−1.87, −0.91], and the superficial processing condition, *M* difference = −0.51, *p* = .032, 95% CI = [−0.98, −0.05]. The magnitude of these differences in change scores from T3 to T4 varied significantly across conditions. The deep processing condition was associated with a larger increase in positive beliefs about the self at recovery relative to exclusion in the deep processing versus superficial processing condition, *t*(250) = −2.59, *M* difference = −0.88, *p* = .011, 95% CI = [−1.53, −0.23], *d* = 0.33. However, at the final recovery time point, participants in the deep processing condition did not differ from participants in the superficial processing condition in their positive self-beliefs ratings, *M* difference = 0.37, *p* = .589, 95% CI = [−0.97, 1.71]. These effects are shown in Figure 2.

#### Effects of condition and time on negative beliefs about others

Analyses revealed a significant change in negative beliefs about others over time, *F*(2.64, 658.92) = 12.367, *p* < .001, η_*p*_^2^ = .05. The main effect of condition was not significant, *F*(1, 250) = 1.301, *p* = .255, η_*p*_^2^ = .01, nor was the interaction between condition and time, *F*(2.64, 658.92) = 1.890, *p* = .138, η_*p*_^2^ = .01. Pairwise comparisons probing the significant main effect of time revealed that negative beliefs about others decreased significantly overall from baseline to initial retrieval, *M* difference = 0.93, *p* < .001, 95% CI = [0.39, 1.48]; then increased significantly after exclusion, *M* difference = −1.02, *p* < .001, 95% CI = [−1.57, −0.46]; and decreased significantly once again after the memory reminder, *M* difference = 0.94, *p* = .001, 95% CI = [0.43, 1.46].

#### Effects of condition and time on positive beliefs about others

Omnibus analyses revealed a significant change in positive beliefs about others over time, *F*(2.85, 714.52) = 45.047, *p* < .001, η_*p*_^2^ = .15. There was no main effect of condition, *F*(1, 256) = .334, *p* = .564, η_*p*_^2^ = .001, but there was a significant Condition × Time interaction, *F*(2.85, 714.52) = 2.720, *p* = .047, η_*p*_^2^ = .01. Follow-up 2 × 2 tests were conducted to probe the significant interaction effect at each phase of the study separately.

At initial retrieval, the Time × Condition interaction was significant, *F*(1, 252) = 7.499, *p* = .007, η_*p*_^2^ = .03. Pairwise comparisons demonstrated that positive beliefs about others increased significantly after initial memory retrieval compared with baseline in the deep processing condition, *M* difference = −1.58, *p* < .001, 95% CI = [−2.14, −1.03], but not in the superficial processing condition, *M* difference = −0.50, *p* = .071, 95% CI = [−1.04, 0.04]. The magnitude of these differences in change scores from T1 to T2 varied significantly across conditions. The deep processing condition was associated with a more robust increase in positive beliefs about others at initial retrieval relative to baseline than the superficial processing condition, *t*(252) = −2.74, *M* difference = −1.08, *p* = .003, 95% CI = [−1.91, −0.34], *d* = 0.34.

At exclusion, the Time × Condition interaction was significant, *F*(1, 252) = 4.388, *p* = .037, η_*p*_^2^ = .02. Pairwise comparisons demonstrated that positive beliefs about others decreased significantly in both deep processing, *M* difference = 2.92, *p* < .001, 95% CI = [2.28, 3.56], and superficial processing, *M* difference = 1.96, *p* < .001, 95% CI = [1.33, 2.59]. The magnitude of these differences in change scores from T2 to T3 varied significantly across conditions. The deep processing condition was associated with a greater decrease in positive beliefs about others at exclusion relative to exclusion than the superficial processing condition, *t*(252) = 2.10, *M* difference = 0.96, *p* = .035, 95% CI = [0.11, 1.84], *d* = 0.34.

At recovery, the Time × Condition interaction was not significant, *F*(1, 251) = .052, *p* = .820, η_*p*_^2^ = .00. Participants experienced similar significant increases in positive self-beliefs at recovery compared with exclusion in both the deep processing condition, *M* difference = −1.30, *p* < .001, 95% CI = [−1.88, −0.72], and the superficial processing condition, *M* difference = −1.21, *p* < .001, 95% CI = [−1.77, −0.65].

### Additional analyses

For analyses examining effects of condition on raters’ objective ratings of the valence and vividness of participants’ memory narratives, see the Supplemental Material.

## Discussion

The current preregistered experimental study is among the first to investigate the nature and impact of positive social autobiographical memory retrieval in people with high levels of SA symptoms. On the basis of a growing literature supporting the benefits of therapeutic procedures for SAD that help patients unhook the self from the past by reappraising the self-relevant meaning of negative autobiographical memories, we reasoned that socially anxious individuals may also benefit from “hooking” the self onto the past by deepening self-relevant appraisals of memories for positive social experiences. We predicted that deep self-relevant processing of the memory—relative to shallow perceptual processing—would facilitate more positive and less negative affective, interpersonal, and cognitive outcomes at initial memory retrieval relative to baseline; protect participants more effectively against the negative effects of exclusion; and aid them in recovering from exclusion more resiliently.

Results demonstrated, first, that high-SA individuals were reliably able to access specific, detailed, positive autobiographical memories in which they felt connected, valued, or accepted by others. Participants’ written narratives across conditions consistently conveyed that the experiences they recollected tended to evoke strong feelings of belongingness and social acceptance. Although many participants reported positive memories of engaging in pleasurable social activities, some participants reported positive experiences of feeling supported and accepted by significant others during a difficult life event. With respect to participants who remembered receiving desired support during a difficult life event, a common theme was a feeling of pleasant surprise or relief upon discovering that others were compassionate and helpful rather than rejecting or critical. These observations are merely qualitative, but they suggest that there may be different types of positive social autobiographical memories in SA that elicit feelings of belongingness and interpersonal security for different reasons. Research has shown that individuals with SAD may respond differently to the same outcomes when framed as the presence of something positive versus the absence of something negative (Alden et al., 2004); thus, it is possible that positive memories of approaching experiences with expected pleasurable outcomes may evoke different responses than those of avoiding experiences with expected negative outcomes. Future research could investigate whether these different types of memories have varying mnemonic, emotional, and interpersonal characteristics that may affect participants’ behavior in different ways when cued within social contexts.

Although we instructed participants to retrieve positive memories that were associated with feeling connected, valued, or accepted by others, it is possible that they may have been able to retrieve other types of positive social autobiographical memories that were not probed in the current study, such as memories of personal accomplishments. Furthermore, we asked them to retrieve only a single positive memory, but it is possible that they may have been able to recall several such memories. Future research should also examine whether a similar capacity to retrieve specific positive autobiographical social memories extends to clinical participants with SAD, especially participants with SAD and comorbid depression, because prior studies have shown that people with clinical depression exhibit deficits in accessing detailed and specific autobiographical memories (Söderlund et al., 2014; Williams et al., 2007), and in benefiting from positive memory retrieval when processed concretely or self-reflectively (Werner-Seidler & Moulds, 2012). We specifically prompted participants in the current study to recollect a specific experience that made them feel socially accepted; however, given prior work showing that people with SAD may be prone to interpreting positive events negatively (Alden et al., 2008) and experiencing elevated anxiety in response to positive evaluative experiences (Weeks & Howell, 2012), it will be essential to pursue future replication and extension of our findings in clinical samples.

The self-report manipulation check revealed that participants believed that although the superficial processing condition facilitated more perceptual processing of the memory than the deep processing condition, the two conditions facilitated similar levels of deep processing, suggesting that the mere act of intentionally retrieving and writing about a positive autobiographical memory in and of itself could promote the perception of deeper processing. However, as illustrated in Table 2, the objective ratings of the narratives themselves told a somewhat different story. These ratings revealed that the deep and superficial processing narratives differed markedly from one another in expected ways with very large effect sizes. Specifically, the narratives produced in the superficial processing condition contained significantly more perceptual details about the elements within the memory scene, whereas participants in the deep processing condition focused significantly more on self-relevant appraisals of the meaning of the experience in relation to how participants viewed their life, their future, and their relationships with others. Additional ratings revealed that the superficial processing narratives were more detailed and vivid, whereas the deep processing narratives were imbued with more positive and negative emotional details.

What impact did the manipulation have on outcomes? Results of primary analyses largely supported our preregistered hypotheses in demonstrating that positive memory retrieval benefited high SA participants most when they were assigned to process their memories deeply by explicitly attaching self-relevant meaning to them. This pattern of results differed somewhat across specific measures: At initial retrieval, participants in the deep processing condition experienced significantly greater increases in positive affect, social safeness, and positive beliefs about others than participants in the superficial processing condition, whereas between-conditions differences were no longer apparent at recovery. Conversely, for negative and positive self-beliefs, participants in the deep processing condition derived significantly greater benefits at recovery than participants in the superficial processing condition even though there were no differences at initial retrieval. Indeed, following the final recovery time point, participants who had processed their positive memories deeply by reflecting on their self-relevant meaning reported significantly reduced negative beliefs about themselves compared with participants who had processed their memories superficially by focusing on the perceptual details within their memory scene. Finally, for negative affect and negative beliefs about others, the condition to which participants were assigned did not moderate the magnitude of changes over time; participants in both conditions reported significant but comparable decreases over time at both initial retrieval and recovery.

These findings provide preliminary support for the use of positive memory retrieval as a positive psychology intervention (e.g., Sin & Lyubomirsky, 2009) that could be intentionally deployed by socially anxious individuals in daily life to enhance subjective well-being across emotional, interpersonal, and cognitive domains. They also support the use of positive memory retrieval as a psychological repair strategy (e.g., Campbell-Sills et al., 2006; Fiksdal et al., 2019) that could help socially anxious individuals achieve more efficient and resilient recovery from stressful social experiences such as ostracism (see Hudd & Moscovitch, 2020, 2023). It is promising that such resilience appears especially tied to benefits for negative and positive beliefs about the self, which are fundamental to the psychopathology of SAD. For people with high SA, memories of being accepted by others, especially when they expect to be rejected, may be particularly powerful tools for challenging persistent negative self-schemas that focus on being socially undesirable (see D. A. Moscovitch et al., 2023). Future studies must examine the longer-term effects of retrieving and deeply processing positive social memories, particularly on self-beliefs, and whether effects generalize to clinical samples of people with SAD in both well-controlled settings, such as the laboratory or clinic, and naturalistic settings within people’s daily lives.

In contrast to the benefits of positive memory retrieval at initial retrieval and during recovery from exclusion, there was no evidence to support the hypothesis that positive memory retrieval helps to inoculate socially anxious individuals against the negative effects of social exclusion by preventing or mitigating its immediate impact (e.g., Meichenbaum & Deffenbacher, 1988; Saunders et al., 1996). The same measures that were associated with larger differential gains at initial retrieval for participants in the deep processing condition relative to participants in the superficial processing condition were also associated with greater losses at exclusion, with more significant deterioration in positive affect, social safeness, and positive beliefs about others following Cyberball for participants assigned to deep processing. These findings suggest that for high SA individuals, the benefits of positive memory retrieval may be fragile and tenuous, requiring repeated practice over time for the effects to become well integrated and consolidated into the autobiographical memory system.

Although all significant Condition × Time interaction effect sizes were small, it is likely that our study design minimized the benefits of deep versus shallow processing because such effects may have been even larger if the deep processing condition was compared with a neutral or inert control condition. Future studies could explore the effects of deep processing compared with alternative control conditions, such as a neutral memory or a positive stimulus that is not self-relevant. Future research could also examine the effects of deep positive memory processing in high-SA participants relative to a low SA control group.

Developing new clinical interventions involves testing whether specific procedures affect specific outcomes through specific change mechanisms (e.g., Bruijniks et al., 2019; Kazdin, 2007). To this end, it would be of interest in future research to evaluate the effectiveness of positive memory retrieval and deep processing as a brief intervention procedure for SAD, especially compared with the IR procedure, which has already been shown to be effective as a single-session intervention in numerous studies (see Romano, Moscovitch, et al., 2020). It is possible that both IR and self-relevant processing of positive memories are procedures that work through similar schema-based learning mechanisms. As detailed in D. A. Moscovitch et al.’s (2023) schema-congruent and -incongruent learning model, research on the neural basis of schema change suggests that updating of schema-based knowledge (e.g., “I am socially undesirable”; “Social situations are threatening”) occurs through the memory-based mechanisms of episodic mental simulation and prediction error (see also Levy & Schiller, 2021; van Kesteren & Meeter, 2020). Although IR, which is designed to modify negative memories, may be a helpful intervention technique when the clinical goal is to weaken a maladaptive schema, positive memory processing may be especially useful when the clinical goal is to strengthen an adaptive schema. Indeed, successfully modifying self-relevant beliefs in response to positive information may require intervention processes that are distinct from those used to facilitate self-updating in response to negative information (see Sharot & Garrett, 2016). As Padesky (1994) noted almost 30 years ago, clinical interventions designed to facilitate schema change often work synergistically by both weakening the influence of negative or maladaptive schemas and strengthening the influence of positive or adaptive ones.^2^

Given that SAD is characterized by both oversensitivity to social threat and undersensitivity to social reward (Hudd & Moscovitch, 2021), clinicians may benefit from having a range of intervention procedures at their disposal to facilitate schema-based learning in patients with SAD to help patients both unhook the self from negative experiences and hook the self onto positive ones (D. A. Moscovitch et al., 2023). Although memory-based interventions that are designed to unhook self-meaning from negative memories may work by down-regulating the threat system and recalibrating negatively biased threat expectations through the process of schema-incongruent learning, those that are designed to hook self-meaning onto positive experiences may work by up-regulating the reward system and recalibrating typically inhibited reward expectations for the self (see Alden & Taylor, 2010; Hudd & Moscovitch, 2022, 2023; Kashdan, 2007). Although we are not aware of any studies that have directly compared the effects and mechanisms of these two types of memory-based interventions in patients with SAD, emerging evidence suggests that treatments for anhedonia designed intentionally to improve positive affect are associated with increases in patients’ sensitivity to rewards and tend to achieve better clinical outcomes than those designed to target negative affect (Craske et al., 2023; see also Kryza-Lacombe et al., 2021; Taylor, Pearlstein, et al., 2020).

The present study was limited by its recruitment of undergraduate participants who were relatively young, largely female, and primarily White/European, East Asian, or South Asian. Although the sample reflected some diversity across other ethnic and cultural groups, future studies on more diverse community-based samples are necessary to examine whether and how individual differences in participants’ demographic characteristics and lived experience may affect positive-memory retrieval and its effects. A related limitation was that our collection of demographic data did not distinguish between racial, ethnic, and cultural categories. Rather, participants were permitted to select “all that apply” from numerous response options that align with those typically used in the Canadian cultural context in which this study was conducted or to enter their own selection in an open text box. Moreover, we did not collect data on participants’ income, education, or socioeconomic status, although future studies (particularly those on community nonstudent samples) should aim to do so. In addition, although study procedures relied on standardized, reliable measures and behavioral tasks, the study itself was conducted exclusively online and requires replication and extension in naturalistic and lab-based environments in which observations of actual behavior could complement self-report assessments. Finally, it is unclear whether or how the context of the COVID-19 global pandemic may have affected participants’ engagement in the study tasks. Data collection occurred from September 2021 to April 2022, during the second year of the pandemic, when many COVID-related restrictions were still commonplace but gradually shifting as vaccinations became available in Canada, where the present study took place.

Despite these limitations, results of the present study significantly advance the field’s understanding of the nature of positive social autobiographical memory retrieval in SA and the conditions under which it is most effective, thereby helping to pave the way for future studies investigating memory mechanisms in the psychopathology and treatment of SAD.

## Supplemental Material

sj-docx-1-cpx-10.1177_21677026231195792 – Supplemental material for Hooking the Self Onto the Past: How Positive Autobiographical Memory Retrieval Benefits People With Social Anxiety Supplemental material, sj-docx-1-cpx-10.1177_21677026231195792 for Hooking the Self Onto the Past: How Positive Autobiographical Memory Retrieval Benefits People With Social Anxiety by David A. Moscovitch, Kendra White and Taylor Hudd in Clinical Psychological Science

## Transparency

*Action Editor:* DeMond M. Grant

*Editor:* Jennifer L. Tackett

*Author Contribution(s)*

**David A. Moscovitch:** Conceptualization; Data curation; Formal analysis; Funding acquisition; Investigation; Methodology; Project administration; Resources; Software; Supervision; Validation; Writing – original draft.

**Kendra White:** Conceptualization; Data curation; Formal analysis; Methodology; Project administration; Writing – review & editing.

**Taylor Hudd:** Conceptualization; Data curation; Formal analysis; Investigation; Methodology; Project administration; Supervision; Visualization; Writing – review & editing.

**Open Practices:** This article has received badges for Open Data, Open Materials, and Preregistration. More information about the Open Practices badges can be found at https://www.psychologicalscience.org/publications/badges

## Notes

1. Our preregistered hypotheses and data analytic plan (https://osf.io/nc3w5) originally subdivided these analyses into two sets of mixed-model ANOVAs, with a first set of 2 × 2 tests examining the effects of condition on outcomes from T1 (baseline) to T2 (after manipulation) and a second set of 2 × 3 tests examining the effects of condition on outcomes across T2 (after manipulation), T3 (after Cyberball), and T4 (recovery). However, for the sake of parsimony, we instead conducted omnibus analyses as one set of 2 × 4 mixed-model analyses across all time points, with significant omnibus effects followed up with 2 × 2 tests examining each set of paired time points separately. Note that none of the preregistered exploratory analyses were conducted for the current project (thus, the additional self-reported measures that were collected for these exploratory analyses are not reported here). Finally, exploratory analyses of coded narrative variables were added post hoc, and we thank an anonymous reviewer for this suggestion.

2. Whether effective exposure-based cognitive-behavioral interventions for emotional disorders work by modifying schemas directly or by altering their strength or accessibility is currently a matter of debate within the clinical literature (for a review, see Huppert et al., 2020). According to retrieval competition theory (Brewin, 2006) and inhibitory learning theory (Craske et al., 2008, 2014), treatment allows patients to gain access to positive mental self-representations, which then compete with the original negative representations for retrieval within subsequent contexts. In contrast, proponents of reconsolidation theory (Ecker, 2020; Elsey et al., 2018) and early versions of emotional processing theory (Foa & Kozak, 1986) argued that effective treatment enables the direct modification of negative mental representations.
