# Supplementary material for: Hooking the Self Onto the Past: How Positive Autobiographical Memory Retrieval Benefits People With Social Anxiety
Source: Clin Psychol Sci. 2023 Nov 15;12(5):882–902. doi: 10.1177/21677026231195792 (PMC11415290; doi:10.1177/21677026231195792)
Supplement: sj-docx-1-cpx-10.1177_21677026231195792 – Supplemental material for Hooking the Self Onto the Past: How Positive Autobiographical Memory Retrieval Benefits People With Social Anxiety [file sj-docx-1-cpx-10.1177_21677026231195792.docx]

**Supplemental Materials**

**1. Rationale for *a priori* sample size estimations**

Our expectation of a medium effect size is consistent with the meta-analysis by Kashdan (2007) on the magnitude of the correlation between SA and positive affect (r = -.36) as well as Alden and Taylor’s (2011) treatment study showing that interpersonal-based cognitive behavioural therapy demonstrated large effect size increases in a composite measure of positive social functioning over time relative to waitlist in patients with SAD (partial eta squared = .20). We oversampled to compensate for expected missing or invalid data and for the possibility that smaller effect sizes may be observed when comparing two active study conditions that both included positive memory retrieval – a technique that has already been shown in prior studies to promote adaptive outcomes in healthy adults as well as people with depression.

**2. Descriptions of memory retrieval prompts**

All participants were instructed to recall a personal memory where they felt accepted, connected, or valued by others. Participants who indicated they were unable to retrieve such a memory were provided with a series of three prompts, as follows: (a) “Think of an important relationship in your life. Now think of a time in your life when you were with that person and felt an intense happiness. Are you able to bring such an experience to mind?”; (b) “Think of a moment within your life when you were around someone important to you and who made you feel comfortable, relaxed, or safe with them. Are you able to bring such an experience to mind?”; and (c) “Think of a time within your life when you were with someone important to you. Now think of a time when this person made you feel appreciated. Are you able to bring such an experience to mind?” Prompts were presented one at a time and participants only progressed to the next prompt if the preceding prompt failed to facilitate the retrieval of a positive social memory.

**3. Instructions for memory retrieval exercise across conditions**

1. *Deep processing condition instructions:*

When you have your memory in mind and you are ready, please visualize yourself in that situation, feeling loved and accepted by others. Focus on your deepest thoughts and feelings within the memory. How did it feel to be in that situation? What kinds of thoughts were you having as you were in that situation? Please begin writing now. (3 mins)

Bring the memory back to mind and focus on the events within the memory until you form a strong image of it. Notice how your needs were satisfied by this experience. How did having your needs met make you feel? How did you behave in ways that helped satisfy your needs during this experience? (3 mins)

Hold the memory in mind and reflect on your actions within the memory and interactions with others. What do you think this experience says or means about you as a person? What allowed you to feel connected to the other people in your memory? (3 mins)

Close your eyes and re-imagine the memory. Once you have brought the memory back to mind, reflect on what this memory tells you about who you are and how other people feel about you? What does this experience mean about your life and your future. (3 mins)

1. *Superficial processing condition instructions:*

When you have your memory in mind and you are ready, close your eyes and visualize yourself in that situation. With the memory in mind, consider the space around you within the memory scene. Where are you located in space and time? Are you indoors or outdoors? Is the environment crowded or open-spaced? What time of day is it? Where are you located relative to others? (3 mins)

Take a moment, to close your eyes and bring the memory to mind again. Pay attention to the details of those around you. Using your imagination, try to count the number of people who are present in the memory. Of this group, how many people had you seen before? Try to imagine and describe what everyone is wearing in as much detail as possible. (3 mins)

Hold the memory in mind, close your eyes and notice the details of the surrounding objects within the memory. Describe any objects you see in the environment within your memory. What are their shapes and sizes?  Is there anything moving? What colours appear in your memory? (3 mins)

Bring the memory back to mind and search for other people within the memory. Choose any two people in your memory scene (including yourself) and spell their first names. Write down as many words as you can think of starting with any one of the letters in each person’s first name. (3 mins)

**4. Research assistant training and additional objective ratings of participants’ memory narratives**

*Training.* Research assistants were trained to use the rating scale to complete the objective manipulation check in a 2-hour initial meeting with the first author during which each RA reviewed and independently rated several practice narratives, one at a time, revealed and justified their ratings to one another, resolved discrepancies through collaborative discussion, and worked together to develop criteria to guide subsequent ratings. Once coding norms were established, RAs were instructed to work independently to rate each narrative one at a time until all memory narratives had been completed. Thus, each narrative was associated with ratings from two independent coders, and these data were subjected to a reliability analysis, which is described below.

*Additional objective ratings: valence and vividness of written narratives.* RA raters also rated each narrative on the following three items on the same 0-4 Likert scale described above: (a) Positive valence: “To what extent does the participant describe their experience as being positive?”; (b) Negative valence: “To what extent does the participant describe their experience as being negative?”; and (c) Vividness: “Overall, to what extent does the participant describe the narrative in a clear and detailed way that provides a vivid image of the memory scene?” Training on how to rate these items followed the same procedures outlined above. Two-way random-effects Intraclass Correlation Coefficients (ICC) were computed for absolute agreement of the average rating (since the average rating between the two raters was our intended unit of analysis), which yielded the following values: (a) Positive valence ICC = .888; (b) Negative valence ICC = .695; (c) Vividness ICC = .659. Based on recommended interpretations of ICC values (Koo & Li, 2016), these values reflect “moderate” (values between 0.5 and 0.75) to “good” (values between 0.75 and 0.9) agreement between raters across these rated outcomes.

As noted in Footnote 1 (main manuscript), coding the narrative data to conduct an objective manipulation check was recommended by an ad-hoc reviewer and not included in our preregistered plan.

**5. Additional analyses: effects of condition on objective ratings of memory valence and vividness**

Objective ratings of how positively participants described their experience was higher in the deep processing condition (*M* = 2.97, *SD* = .63) relative to superficial processing (*M* = 1.33, *SD* = 1.04), *t*(253) = 15.17, *Mdiff* = 1.64, *p* < .001, 95%CI [1.44, 1.86], *d* = 1.90. Ratings of how negatively participants described their experience was also higher among those assigned to the deep processing condition (*M* = .97, *SD* = .84) relative to superficial processing (*M* = .41, *SD* = .72), *t*(253) = 3.88, *Mdiff* = .38, *p* < .001, 95%CI [.18, .57], *d* = .49, though both conditions were associated with near-floor ratings of negativity, as expected for appraisals of positive memories. Finally, raters judged that participants in the superficial processing condition provided more vivid memory narratives (i.e., containing clearer details that provided a vivid picture of the memory scene) (*M* = 1.80, *SD* = .72) than those in the deep processing condition (*M* = .72, *SD* = .71), *t*(253) = -13.47, *Mdiff* = -1.08, *p* < .001, 95%CI [-1.25, -.93], *d* = 1.69.

As noted above and in Footnote 1, these additional ratings were not included in our preregistered plan.
